# Polyarticular Septic Arthritis Following Hemodialysis in an Immunocompetent Patient With Acute Renal Failure

**DOI:** 10.7759/cureus.110632

**Published:** 2026-06-10

**Authors:** Sidhant Talwar, Christopher J Pinto, Mayur Bhat

**Affiliations:** 1 Internal Medicine, Vijayanagar Institute of Medical Sciences, Bellary, IND; 2 Family Medicine, Western Michigan University Homer Stryker M.D. School of Medicine, Kalamazoo, USA

**Keywords:** emergency medicine, infectious disease, nephrology, orthopedics, tropical medicine

## Abstract

A man in his early 50s with no significant prior renal history was admitted with acute kidney injury and sepsis secondary to right lower limb cellulitis, requiring prompt multidisciplinary management and further evaluation of the underlying etiology. Initial treatment included hemodialysis, following which the patient developed polyarticular septic arthritis (PASA), a rare condition. Examination revealed significant swelling and tenderness in multiple joints, and microbiological analysis confirmed a *Staphylococcus aureus* infection. Hemodialysis precipitated the bloodstream infection, as blood cultures were negative before hemodialysis but positive for *Staphylococcus aureus* post-dialysis. The patient underwent multiple joint washouts and was treated with intravenous vancomycin and linezolid. The patient’s renal function normalized after treatment, and his symptoms resolved without recurrence. This case highlights the potential for PASA in an immunocompetent individual after a single hemodialysis session, emphasizing the role of catheter-related bloodstream infection (CRBSI) in its pathogenesis and underscoring the importance of timely diagnosis and treatment to prevent severe outcomes. Such presentations underscore the importance of heightened clinical vigilance, as delayed recognition can result in significant morbidity.

## Introduction

Septic arthritis is defined as inflammation of the joint cavity secondary to infection. Distribution is usually monoarticular (85-90%) and typically involves large joints (knee, hip) [1,2]. However, polyarticular involvement, although rare, is associated with greater morbidity, diagnostic difficulty, and higher mortality rates [3-5]. Polyarticular septic arthritis (PASA) may mimic inflammatory arthropathies and thus require a high index of clinical suspicion and prompt evaluation [3,4]. Patients undergoing hemodialysis are especially predisposed due to a combination of impaired cellular and humoral immunity and frequent exposure to vascular access [2,6]. Although multiple large joint involvement is rarely seen, our patient developed PASA following a hemodialysis session for acute renal failure secondary to cellulitis and sepsis.

## Case presentation

**Figure 1:**
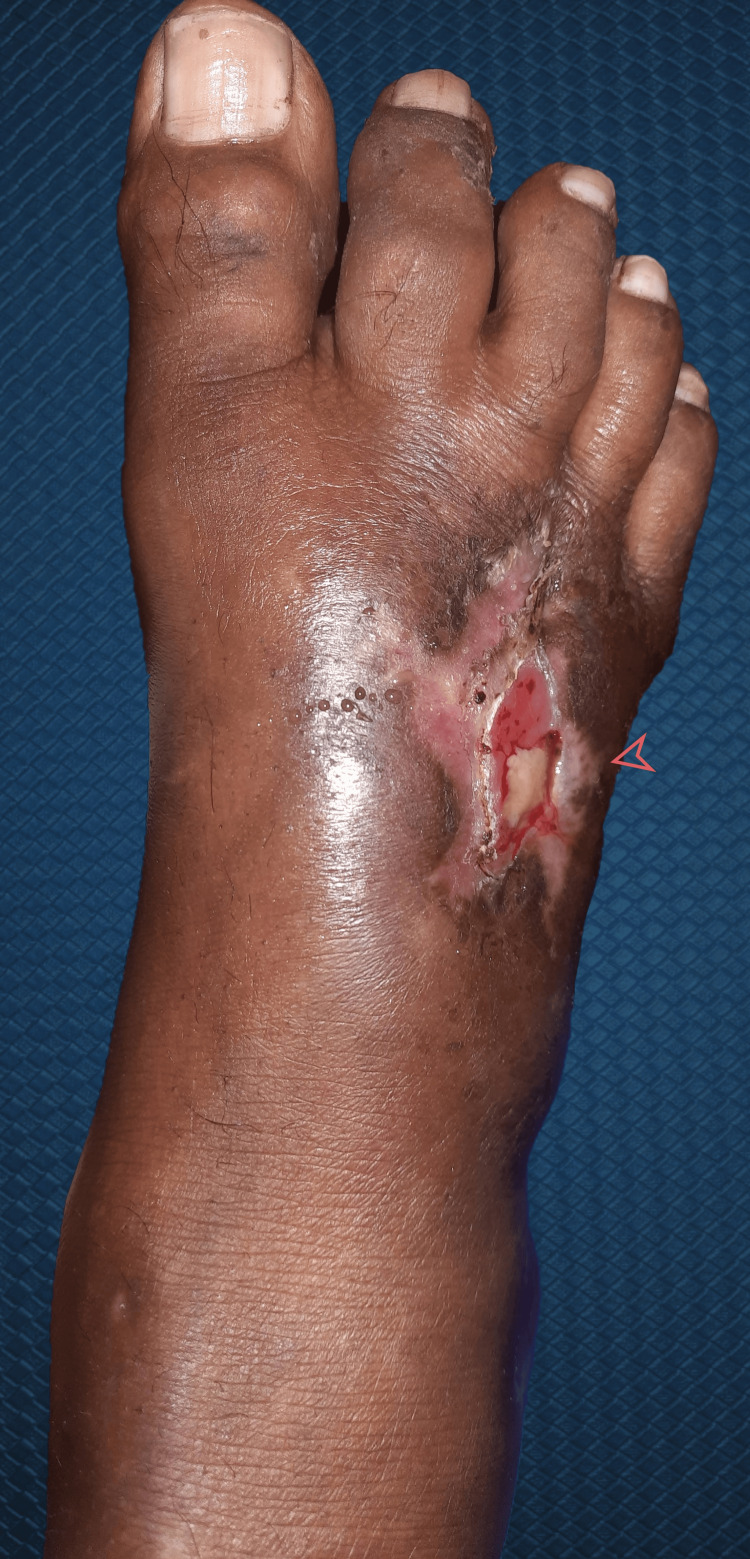
Physical examination of the dorsal right foot showing a wound with edematous peripheral subcutaneous tissue indicating cellulitis Dorsal right foot wound with surrounding soft-tissue edema and erythema, indicative of cellulitis. The lesion served as the primary infectious focus contributing to the patient's clinical presentation.

A man in his early 50s presented to the emergency department with complaints of decreased urine output for three days and suture dehiscence of the wound over his right lower limb for five days (Figure 1). There was no history of steroid or drug use. He denied alcohol usage and smoking. He had no history of diabetes, hypertension, or any other comorbidity. On examination, the patient was febrile (38.9℃), with tachycardia (109/min), and had a blood pressure of 74/45 mmHg. There was significant swelling and tenderness to palpation over the right foot.

Blood investigations showed mild anemia, leukocytosis, and raised inflammatory markers. The peripheral smear demonstrated normocytic normochromic anemia with a few microcytes. HIV and hepatitis serology (hepatitis B and C) were both negative. The routine urine showed trace albumin and 1-2 pus cells/hpf. Autoimmune workup, including ANA, ANCA, and rheumatoid factor, was negative. The investigations have been summarized in Table 1. An elevation of the patient's baseline creatinine recorded three months prior from 0.9 mg/dL to 6.8 mg/dL in conjunction with anuria was suggestive of acute renal failure. The anemia was attributed to poor nutritional status. The elevated BUN-to-creatinine ratio was suggestive of prerenal failure. The initial blood culture was sterile.

**Table 1:** Summary of hematological and urinary investigations TLC: total leukocyte count, SGOT: serum glutamic-oxaloacetic transaminase, SGPT: serum glutamic pyruvic transaminase, ALP: alkaline phosphatase, HIV: human immunodeficiency virus, ESR: erythrocyte sedimentation rate, CRP: C-reactive protein, PCR: protein creatinine ratio

Investigation	Results	Reference range
Hemoglobin	11.3 g/dL (L)	13.2-16.6 g/dL (male)
TLC	14.77 x 10^9^/L (H)	4-11 x 10^9^/L
Platelets	215 x 10^9^ /L	150-400 x 10^9^ /L
Total bilirubin	1.1 mg/dL	0.3-1.3 mg/dL
Indirect bilirubin	0.3 mg/dL	0.2-0.4 mg/dL
SGOT	34 U/L	12-38U/L
SGPT	38 U/L	7-41U/L
ALP	312 IU/L (H)	44-147 IU/L
Urea	129 mg/dL (H)	5-45 mg/dL
Creatinine	6.8 mg/dL (H)	0.6-1.4 mg/dL
BUN	153 mg/dL (H)	6-24 mg/dL
BUN-to-creatinine ratio	22.9:1, indicative of prerenal failure	10:1-20:1
Sodium	135 mmol/L	135-145 mmol/L
Potassium	6.1 mmol/L (H)	3.5-5.5 mmol/L
HbA1c	5.40%	<5.7%
Uric acid	4.1 mg/dL	3.5-7.5 mg/dL
ESR	92 mm/hr (H)	0-30 mm/hr
CRP	85 mg/dL (H)	<10 mg/dL
Procalcitonin	17 µg/L (H)	0-0.05 µg/L
C3	131.1 mg/dL	80-160 mg/dL
C4	43.9 mg/dL	15-45 mg/dL
Anti-CCP	<7 EU/mL	<20 EU/mL
Urine PCR	1.28 mg/ mmol	<3 mg/mmol

**Figure 2:**
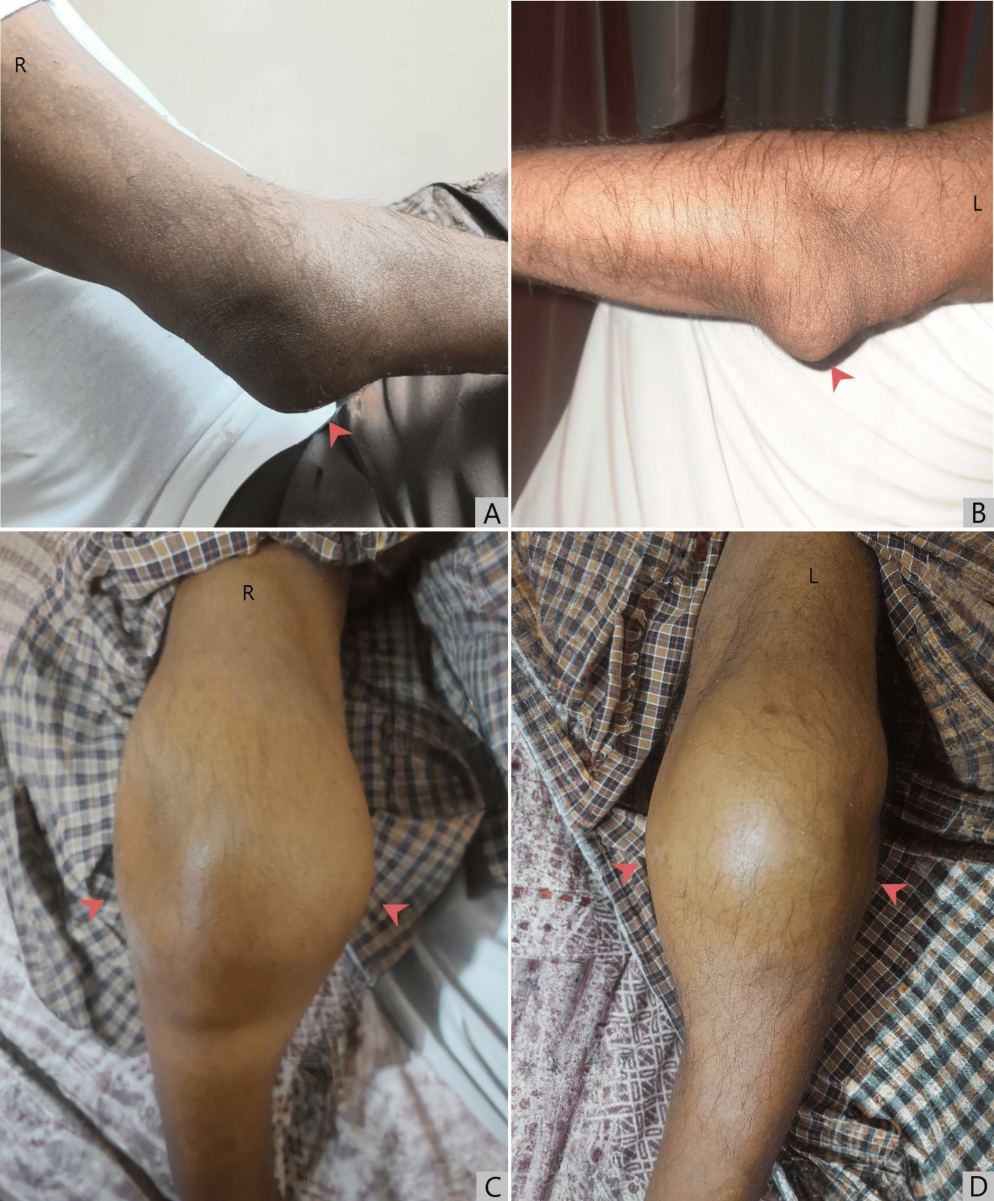
Physical findings of the upper and lower extremities highlighting visible large joint effusions Physical examination showing bilateral elbow (A-B) and bilateral knee (C-D) joint effusions. Polyarticular large-joint involvement is consistent with the patient's diagnosis of PASA. PASA: polyarticular septic arthritis

**Figure 3:**
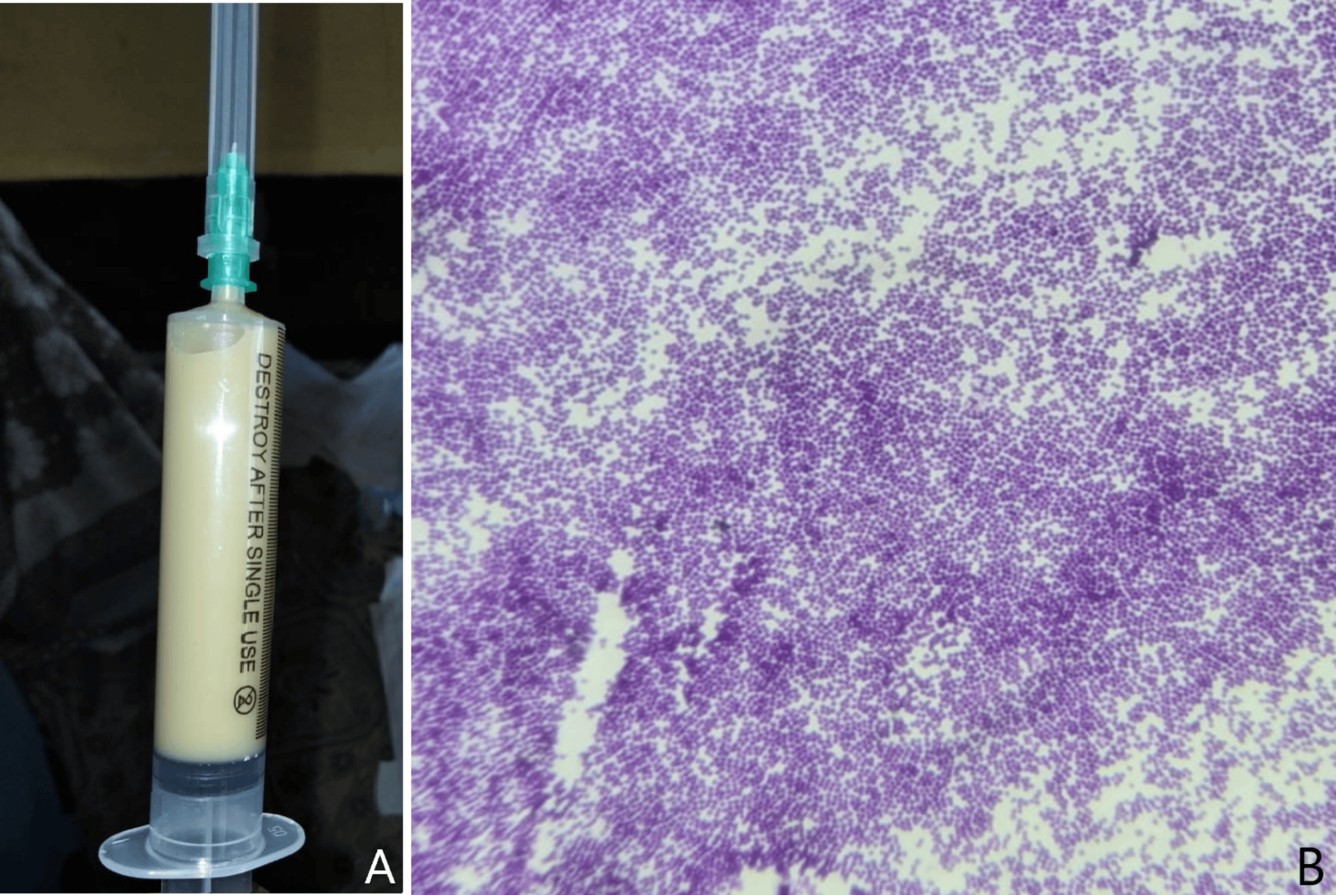
(A) Fluid from knee joint aspiration. (B) Gram staining of the joint aspiration showing gram-positive Staphylococcus aureus in clusters Gram-stained synovial fluid smear (×1000, oil immersion) showing Gram-positive cocci in clusters, consistent with *Staphylococcus aureus* and confirming the infectious etiology of the patient's septic arthritis.

The patient was diagnosed with sepsis secondary to cellulitis, leading to acute renal failure. Given the reduced urine output (<50 ml/24 h), azotemia, and anemia, the patient underwent two sessions of hemodialysis via the right internal jugular catheter. On the third day of admission, raised temperatures were noted again, with a slight improvement in kidney function. On the fourth day, bilateral knee joint swelling was reported, which was progressive (Figure 2). Ultrasound of his inflamed joints showed effusions with internal echoes, suggestive of septic arthritis. The knee and elbow joints were aspirated and washed out on three separate days by the orthopedic team (Figure 3).

**Figure 4:**
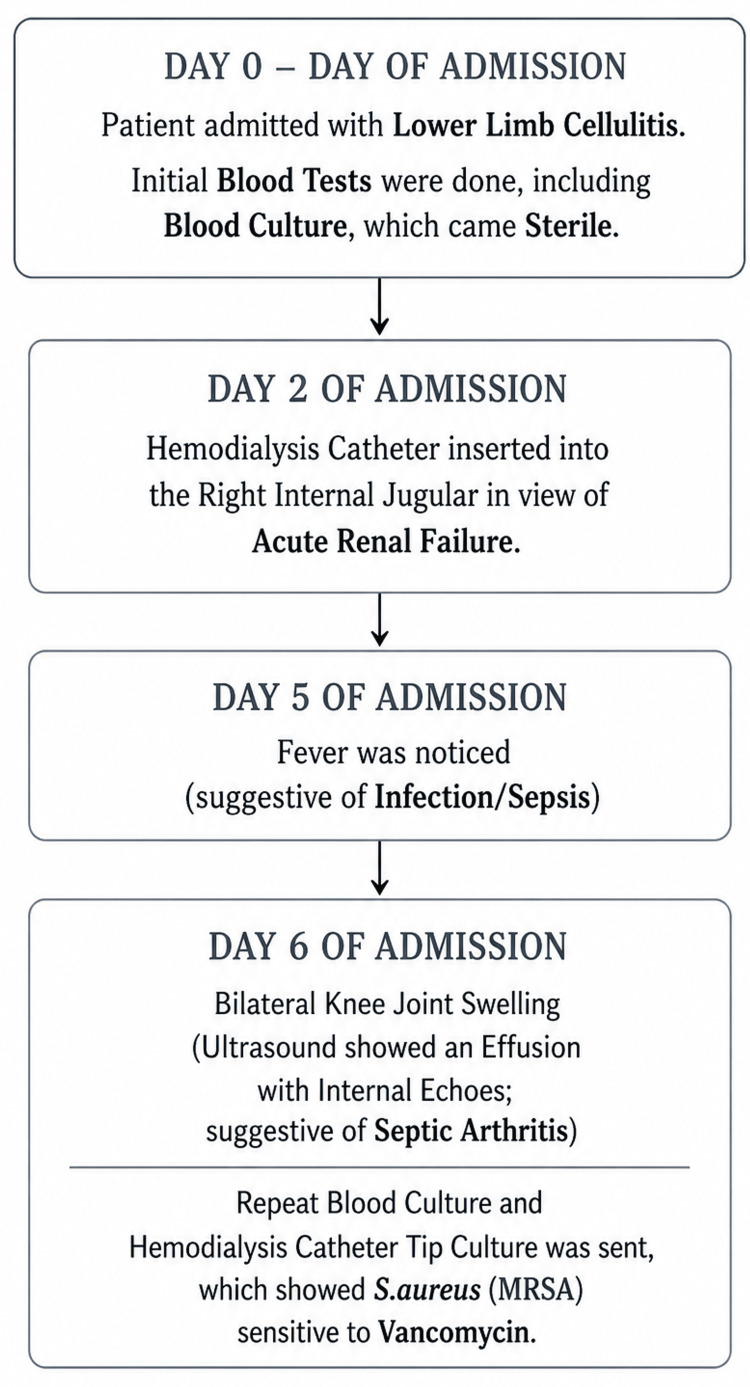
Flowchart of the timeline has been included to summarize key clinical events, interventions, and corresponding cultures' reports MRSA: methicillin-resistant *Staphylococcus aureus*

Synovial fluid was turbid in appearance; a Gram stain showed occasional Gram-positive cocci. Microbiological samples from each washout procedure grew *Staphylococcus aureus*, which was sensitive to vancomycin and linezolid. The indwelling central line was removed, and the tip was also positive for *Staphylococcus aureus*. The blood cultures, which were initially negative at admission, were repeated and were positive for *Staphylococcus aureus* following the hemodialysis session, and the isolate was sensitive to vancomycin. 2D echocardiography and chest X-ray showed unremarkable findings. A flowchart of the timeline is shown in Figure 4.

Although the temporal association between catheter insertion, subsequent positive blood cultures, and growth of the same organism from the catheter tip suggests a catheter-related bloodstream infection (CRBSI) as the most likely source of bacteremia, an alternative possibility is that the pre-existing right foot cellulitis served as the initial focus. Since blood cultures were negative at presentation and cultures from the foot wound were not obtained, a definitive determination of the primary source was not possible. Nevertheless, the microbiological findings and chronology of events strongly supported a significant contribution of the hemodialysis catheter to the development of *Staphylococcus aureus* bacteremia and subsequent PASA.

He was treated with IV vancomycin and linezolid for the next two weeks. Following a recovery in his creatinine levels to 1.0 mg/dL, discharge, and appropriate wound care for the foot wound, his symptoms did not resurface during the follow-up period. Investigations on the day of discharge have been summarized in Table 2. There was significant improvement in range of motion, with a healing scar present over the foot.

**Table 2:** Summary of hematological investigations on day of discharge TLC: total leukocyte count, BUN: blood urea nitrogen, ESR: erythrocyte sedimentation rate, CRP: C-reactive protein

Investigation	Results	Reference range
Hemoglobin	11.2 g/dL (L)	13.2-16.6 g/dL (male)
TLC	6.23 x 10^9^/L (H)	4-11 x 10^9^/L
Platelets	314 x 10^9^ /L	150-400 x 10^9^ /L
Urea	36 mg/dL (H)	5-45 mg/dL
Creatinine	1 mg/dL (H)	0.6-1.4 mg/dL
BUN	16.8 mg/dL (H)	6-24 mg/dL
BUN-to-creatinine ratio	16.8:1	10:1-20:1
Sodium	136 mmol/L	135-145 mmol/L
Potassium	3.9 mmol/L (H)	3.5-5.5 mmol/L
Uric acid	3.3 mg/dL	3.5-7.5 mg/dL
ESR	34 mm/hr (H)	0-30 mm/hr
CRP	16 mg/dL (H)	<10 mg/dL
Procalcitonin	<0.05 µg/L (H)	0-0.05 µg/L

## Discussion

The incidence of septic arthritis in the general population is 4-6 per 100,000 cases annually [7]. There are multiple risk factors for PASA, including diabetes, rheumatoid arthritis, previous joint/skin infection, recent joint surgery, trauma, osteoarthritis, and kidney disease [1,7]. Our patient did not have any of these rheumatologic or immunocompromising factors. Yet, he had acute renal failure and an indwelling catheter, both of which significantly increase the risk of bacteremia and joint infection [2,6]. The literature on PASA in immunocompetent individuals is limited.

The prevalence of CRBSI is around 1.1-5.5 episodes per 2.7 years of catheterization [8]. Our patient had a catheter in place for only a short duration. While tunneling dialysis catheters inserted into the neck vein is a method aimed at reducing the incidence of CRBSI, the risk of infection remains a concern [8,9]. A delay in diagnosis of PASA may lead to significant mortality (7-15% despite treatment), which may be higher if the causative organism is *Staphylococcus aureus* (>50%) [1,10]. Furthermore, repeated vascular access and uremia-associated immune dysfunction in dialysis patients predispose them to severe systemic infections and septic arthritis [6,11].

PASA remains a diagnostic challenge because it can mimic inflammatory arthropathies such as rheumatoid arthritis, crystal arthropathy, or reactive arthritis [3,12]. The presence of fever, elevated inflammatory markers, and rapidly progressive joint symptoms should prompt urgent synovial fluid aspiration and analysis. Imaging modalities such as ultrasound and MRI may aid early detection, especially when clinical findings are subtle [3,4]. Early initiation of targeted intravenous antibiotics together with timely joint drainage remains the mainstay of management and is associated with improved functional recovery and reduced mortality [12,13].

Peri et al. reported a case of polyarticular sepsis following acute hemodialysis in which vascular access-related bacteremia was implicated as the source of infection [9]. Similarly, studies involving the dialysis population have demonstrated a higher incidence of septic arthritis and poorer clinical outcomes compared with the general population, largely attributable to repeated vascular access, frequent healthcare exposure, and impaired host immune responses [2,6]. However, unlike many previously reported cases that occurred in patients with comorbidities or long-term dialysis dependence, this case did not have any underlying immunocompromising condition. It developed PASA after only a brief period of hemodialysis access.

The source of bacteremia in this patient requires careful consideration. The patient initially presented with right foot cellulitis, a recognized source of bacteremia. However, the initial blood cultures obtained on admission were sterile, whereas repeat blood cultures obtained after hemodialysis were positive for *Staphylococcus aureus*. Furthermore, the culture of the removed hemodialysis catheter tip yielded the same organism. Thus, the timing of catheter placement, fever onset, and PASA development indicates that a CRBSI is the most likely source. However, since foot wound cultures were unavailable and cellulitis occurred before catheter insertion, the possibility that the skin and soft-tissue infection was the primary source cannot be entirely ruled out. Therefore, it is plausible that the cellulitis and vascular access both contributed to the infectious process. This case highlights the importance of evaluating both pre-existing soft-tissue infections and indwelling vascular devices in patients who develop PASA. It also emphasizes the importance of early detection and treatment to prevent morbidity and mortality.

## Conclusions

This case highlights the development of PASA in an otherwise immunocompetent patient following short-term hemodialysis for acute renal failure. The clinical presentation of PASA may overlap with that of inflammatory arthropathies, leading to delayed recognition and treatment. Therefore, high clinical suspicion is required when patients present with fever and acute polyarthritis, especially in the setting of recent hemodialysis or indwelling vascular access. It also illustrates the diagnostic difficulty of identifying foci of infection in a patient with cellulitis after hemodialysis. Prompt microbiological sampling, including early synovial fluid analysis and cultures, is essential to prevent irreversible joint damage and systemic complications. Additionally, this case emphasizes the importance of a coordinated multidisciplinary approach involving nephrology, infectious disease, and orthopedic teams to optimize diagnostic accuracy, source control, and overall patient outcomes.
